# Mesenchymal Stem Cells Enhance Colonic Anastomotic Repair Through Augmented Collagen Deposition and Decreased Inflammation in a Rat Model

**DOI:** 10.3390/medsci14020316

**Published:** 2026-06-14

**Authors:** Alexandra Caziuc, Emoke Pall, Andras-Laszlo Nagy, David Andras, Oana Antal, Radu Alexandru Ilies, Lorena Maria Hantig, Aurel Mironiuc, George Calin Dindelegan

**Affiliations:** 1 Department of General Surgery, “Iuliu Hatieganu” University of Medicine and Pharmacy, 400012 Cluj-Napoca, Romania; 2 First Surgical Unit, Emergency County Hospital Cluj, 400006 Cluj-Napoca, Romania; 3 Faculty of Veterinary Medicine, University of Agricultural Sciences and Veterinary Medicine, 400372 Cluj-Napoca, Romania; 4 Department of Biomedical Sciences, Ross University School of Veterinary Medicine, Basseterre P.O. Box 334, Saint Kitts and Nevis; 5 Department of Anaesthesia and Intensive Care, “Iuliu Hațieganu” University of Medicine and Pharmacy, 400012 Cluj-Napoca, Romania; 6 Faculty of Medicine, “Iuliu Hatieganu” University of Medicine and Pharmacy, 400012 Cluj-Napoca, Romania; 7 Second Surgical Unit, Emergency County Hospital Cluj, 400006 Cluj-Napoca, Romania

**Keywords:** anastomosis, leakage, colorectal surgery, stem cells, hydroxyproline

## Abstract

**Background/Objectives**: Mesenchymal stem cells (MSCs), due to their regenerative and multipotent properties, have emerged as promising therapeutic agents in tissue repair and regeneration. These biological characteristics might contribute to optimized anastomotic healing and to a reduction in postoperative complications following digestive surgery. The present study aimed to evaluate whether intraperitoneal or perianastomotic administration of MSCs provides superior healing outcomes in colonic anastomoses in Wistar rats. **Methods**: MSCs were isolated from inguinal adipose tissue harvested from 2 Wistar rats. Thirty male Wistar rats were allocated to 3 groups: (i) the control group, with regular anastomosis, (ii) peri-anastomotic injection of MSCs, and (iii) intraperitoneal injection of MSCs. The animals were sacrificed on postoperative day 14. The evaluated outcomes included clinical evolution, adhesion index, histological characteristics, and tissue hydroxyproline content. **Results**: The incidence of anastomotic leakage and the mortality rate were 0%. Therefore, the present study primarily demonstrates changes in surrogate markers of healing, including inflammatory response, collagen deposition, adhesion formation, and hydroxyproline content. The adhesion index was similar in the groups receiving MSC administration (*p* = 0.05); however, intraperitoneal administration demonstrated superior outcomes when compared to standard anastomosis in reducing adhesion formation (*p* = 0.002). Histopathological analysis showed a decreased inflammatory process and an increased collagen deposition at the anastomotic site following MSC administration (*p* < 0.05). Moreover, tissue hydroxyproline levels were significantly increased after both perianastomotic (0.831 ± 0.02, *p* < 0.05) and intraperitoneal (0.54 ± 0.02, *p* < 0.05) MSC administration compared with the control group (0.251 ± 0.006). **Conclusions**: These results suggest that MSC administration may improve histological and biochemical markers associated with colonic anastomotic healing in a non-ischemic experimental model. The experimental model used is suitable for further studies aimed at determining the optimal indications, routes of administration, and adjunctive agents that may potentiate the effects of MSCs.

## 1. Introduction

Despite the increased knowledge in surgical technique and intensive care, the complications following digestive anastomoses remain an important aspect of colorectal surgery. Anastomotic leakage increases the duration of hospitalization, the costs and alters the prognosis [1,2,3]. Several attempts to improve the outcome of digestive anastomosis by new suture materials, sealants, oxygen supplementation [4], growth factors or other biological substances (adrenomedullin, mesalamine, ascorbic acid) have failed [5,6,7,8].

Based on the multipotent properties of mesenchymal stem cells (MSCs), numerous studies have investigated their role in digestive anastomosis healing. Locally transplanted cell therapy improved all the healing parameters in rat ischemic experimental models [9,10]. By contrast, adipose-derived MSC suture loading showed no effect on anastomotic healing [11]. Although a possible beneficial effect of using MSCs for reducing the complications and increasing resistance of ischemic digestive anastomoses was suggested, there is no clear evidence indicating that MSC administration reduces the incidence of postoperative gastrointestinal leaks in non-ischemic conditions [10]. The results obtained in vitro are also contradictory due to different methods of administration (anastomotic injection or suture loading), the experimental models used and quantification of the effect.

The main objective of our study is to establish the effect of perianastomotic and intraperitoneal injection of MSCs on a reproducible non-ischemic colonic anastomosis model on Wistar rats. Our experimental model offers a particular and objective method for measuring in vivo the role of MSCs in anastomotic healing. In addition, this model allows the association of macroscopic, histopathological, and biochemical parameters involved in tissue repair, thereby offering a comprehensive evaluation of the regenerative potential of MSC therapy. By comparing different routes of administration, this study also aimed to identify the most efficient delivery strategy for optimizing anastomotic integrity and reducing postoperative complications. Furthermore, the findings might contribute to the development of novel regenerative approaches with potential translational applicability in colorectal and digestive surgery.

It is important to note that non-ischemic experimental anastomotic models, like the one used in the current study, are characterized by a low baseline incidence of leakage and are therefore primarily suited for evaluating biological and histological parameters of healing rather than clinically relevant endpoints such as anastomotic leak prevention.

To our knowledge, this study is among the few providing a detailed analysis of colonic anastomotic healing at postoperative day 14 in a rat model. The reason behind the selection of postoperative day 14 was to allow the assessment of later stages of healing, such as collagen maturation, extracellular matrix remodeling and stabilization of the anastomotic site. These processes are not fully captured during the early postoperative inflammatory phase (when the majority of leaks occur).

## 2. Materials and Methods

### 2.1. Experimental Design and Animal Model

Male Wistar rats aged 10 weeks were used in the current study. The animals were housed individually under standard laboratory conditions, with free access to water and standard rodent chow throughout the experimental period. All experimental procedures were conducted in accordance with the Guide for the Care and Use of Laboratory Animals and complied with the provisions of the European Union Directive 2010/63/EU regarding the protection of animals used for scientific purposes. The experiment was approved by the Ethics Committee of “Iuliu Hatieganu” University of Medicine and Pharmacy, Cluj-Napoca, Romania (Approval no. 363/21.10.2014).

### 2.2. Harvesting of Mesenchymal Stem Cells

Rat mesenchymal stem cells (MSCs) were isolated from inguinal adipose tissue (which was harvested from two male Wistar rats). The collected tissue was exposed to enzymatic digestion using collagenase type I (2 mg/mL, Sigma-Aldrich, USA) at 37 °C for 30 min. The resulting cell suspension was filtered through a 70 μm cell strainer (Falcon, Corning Inc., USA) and centrifuged for 5 min at 1500 rpm. The cell pellet was then resuspended in phosphate-buffered saline (PBS) and centrifuged again under the same conditions.

Following quantification, a final concentration of 1 × 10^6^ cells was cultured in Dulbecco’s Modified Eagle Medium (DMEM; Gibco, Thermo Fisher Scientific, USA), supplemented with 15% fetal bovine serum (FBS; Sigma-Aldrich, USA), L-glutamine (2 mM; Gibco, USA), and 1% antibiotic–antimycotic solution (Gibco, USA). Subculturing was realized at approximately 80% confluence.

To verify mesenchymal stem cell characteristics according to the criteria established by the International Society for Cellular Therapy (ISCT), the multipotent differentiation capacity of the isolated cells was assessed [12]. The ability to differentiate into osteogenic, chondrogenic, and adipogenic lineages was confirmed.

For osteogenic differentiation, cells were incubated in osteogenic induction medium consisting of DMEM supplemented with 10 mM β-glycerophosphate (Sigma-Aldrich, USA), 0.2 mM L-ascorbic acid-2-phosphate (Sigma-Aldrich, USA), 100 nM dexamethasone (Sigma-Aldrich, USA), 20% FBS (Hyclone, Cytiva, USA), 100 U/mL penicillin, and 0.1 mg/mL streptomycin (Sigma-Aldrich, USA).

For chondrogenic differentiation, cells were cultured in DMEM supplemented with 10% FBS (Hyclone, USA), 100 U/mL penicillin, 0.1 mg/mL streptomycin (Sigma-Aldrich, USA), and 10 ng/mL transforming growth factor beta-1 (TGF-β1; Sigma-Aldrich, USA).

For adipogenic differentiation, cells were cultured in DMEM supplemented with 10% FBS (Hyclone, USA), 1 µM dexamethasone (Sigma-Aldrich, USA), 0.5 mM isobutylmethylxanthine (IBMX; Sigma-Aldrich, USA), 10 µg/mL insulin (Sigma-Aldrich, USA), 1% L-glutamine, and 1% antibiotic–antimycotic (Gibco, USA).

Differentiation capacity was confirmed using histochemical staining with Alizarin Red (osteogenesis), Alcian Blue (chondrogenesis), and Oil Red O (adipogenesis).

Even though trilineage differentiation (osteogenic, chondrogenic, and adipogenic) confirmed mesenchymal characteristics, immunophenotypic characterization using flow cytometry for standard MSC surface markers was not performed.

### 2.3. Experimental Groups

Thirty male Wistar rats were randomly assigned to one of three experimental groups (*n* = 10 per group). Group A (control group) underwent standard colonic anastomosis without any additional treatment. Group B received a perianastomotic injection of MSCs at the site of the colonic anastomosis. Group C received an intraperitoneal injection of MSCs.

All animals were sacrificed on postoperative day 14 for outcome assessment.

### 2.4. Surgical Intervention

A trained researcher performed all surgical procedures according to a modified protocol described by Ceran et al. [13]. After induction of anesthesia with ketamine 2% (50 mg/kg) and xylazine 10% (5 mg/kg), all animals were shaved and the surgical field was disinfected under sterile conditions. Access to the peritoneal cavity was obtained via a 3 cm midline laparotomy, and the cecum was exposed. The colon was transected completely, approximately 5 cm distal to the cecum.

In each group, a colonic end-to-end anastomosis was performed under ×10 microscopic magnification in a single-layer technique using interrupted 7-0 polydioxanone sutures.

Animals in group A (control) received no additional treatment. In group B, a suspension containing 1 mL of MSCs at a concentration of 1 × 10^6^ cells/mL was administered via perianastomotic injection at two points on the anterior and posterior bowel wall, both proximal and distal to the anastomotic site. In group C, 1 mL of the same MSC suspension (1 × 10^6^ cells/mL) was administered intraperitoneally, distributed in the subdiaphragmatic region, beneath the liver, and between the intestinal loops.

The abdominal wall was closed using a continuous suture of 3-0 polyglactin, and the skin was closed with interrupted 2-0 silk sutures. Postoperatively, animals were given access to water after 12 h and food after 24 h, without administration of additional medications.

### 2.5. Follow-Up

Each animal was attributed an individual tracking sheet to monitor postoperative evolution, including recovery of intestinal transit, occurrence of complications, and body weight dynamics (baseline weight versus weight on postoperative day 14). The difference between day 14 and day 0 body weight was calculated for each subject.

### 2.6. Outcome Assessment

A midline relaparotomy was performed at the end of the study period. The peritoneal cavity was explored by a blinded pathologist. Macroscopic evaluation of the anastomotic site included assessment of stenosis, leakage, and abscess formation.

Adhesion formation was evaluated in a blinded manner using the scoring system described by van der Ham et al. [14]: 0 = absence of adhesions; 1 = minimal adhesions, mainly between the anastomosis and omentum; 2 = moderate adhesions involving the omentum and either the anastomotic site or adjacent small bowel loops; 3 = severe and extensive adhesions, including the formation of an abscess.

### 2.7. Histological Analysis

For histological evaluation, 1 cm colonic segments containing the anastomosis were harvested. The mesenteric one-third of each sample was separated for further analysis. The remaining tissue was fixed in 10% buffered neutral formalin, embedded in paraffin, and sectioned at 4 μm thickness.

Sections were stained using hematoxylin and eosin (H&E) and Goldner’s trichrome method. Slides were examined using an Olympus BX51 microscope (Olympus, Japan), and images were captured with an Olympus DP25 digital camera and processed using Olympus Cell B image acquisition software (Olympus Cell B, Japan).

Histological assessment was performed by a blinded pathologist. A semi-quantitative scoring system with four grades (0–3) was used to evaluate inflammation, collagen deposition, epithelialization, and necrosis: 0 = absent; 1 = rare inflammatory cells, minimal collagen fibers, poor epithelialization; 2 = moderate inflammatory infiltration, moderate fibrosis, incomplete epithelialization; 3 = abundant inflammatory infiltrate, marked fibrosis, and complete epithelialization (adapted after [13] and [15]). Tissue evaluation was performed on randomly selected areas within the anastomotic region.

Although histological scores were ordinal, they were treated as continuous variables for comparative statistical analysis, in accordance with several experimental studies that were previously published.

### 2.8. Hydroxyproline Assay

Mesenteric tissue samples (one-third segment) were used for the quantification of hydroxyproline content, following a modified protocol by Jamal et al. [16]. Hydroxyproline concentration was determined using a Hydroxyproline Assay Kit (Sigma-Aldrich, USA) and expressed as μg/mg tissue, serving as an indirect marker of collagen content.

Samples were homogenized using a Potter-type glass homogenizer and centrifuged at 1500 rpm for 5 min. Supernatants were hydrolyzed by adding an equal volume of hydrochloric acid for 3 h. Absorbance was measured at 560 nm using a spectrophotometer, and hydroxyproline concentration was calculated accordingly.

Hydroxyproline values were normalized to wet tissue weight and expressed as μg/mg tissue.

### 2.9. Statistical Analysis

Statistical analysis was performed using R Commander software and Microsoft Excel (Data Analysis ToolPak).

Data distribution was evaluated using the Shapiro–Wilk test. All continuous variables were analyzed using one-way analysis of variance (ANOVA), followed by Tukey’s post hoc test for multiple pairwise comparisons. Even though histological scores were based on a semi-quantitative ordinal scale (0–3), they were regarded as continuous variables for statistical analysis. This approach is commonly applied in experimental biomedical research, such as cases when ordinal scales approximate interval-level data (particularly in balanced study designs with comparable group sizes). Additionally, parametric tests such as one-way ANOVA are considered robust to mild deviations from normality, especially when normality is confirmed and variances are similar between groups. Because score distributions approximated normality and group sizes were equal across study arms, parametric testing was considered acceptable.

Results were expressed as mean ± standard deviation (SD). A *p*-value < 0.05 was considered statistically significant.

## 3. Results

### 3.1. General Outcomes

Statistical analysis for continuous variables in this section was performed using one-way ANOVA, followed by Tukey’s post hoc test for multiple comparisons.

**Figure 1:**
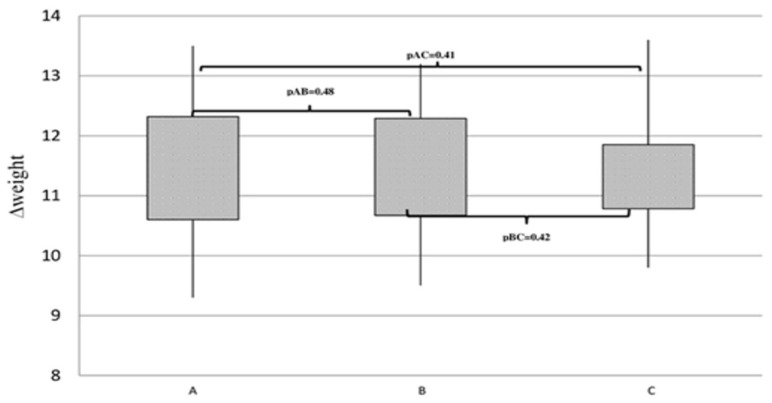
The evolution of body weight across the three experimental groups, expressed as the individual weight difference between postoperative day 14 and baseline (Δweight = weight day 14 − weight day 0). pAB = 0.48, pAC = 0.41, and pBC = 0.42 represent the *p*-values obtained from the comparative statistical analysis between groups A and B, groups A and C, and groups B and C, respectively. Data are presented as mean ± SD. Statistical comparisons were performed using one-way ANOVA with Tukey’s post hoc test.

The postoperative evolution was favorable in all experimental groups. The incidence of anastomotic leakage and the 2-week mortality rate were both equal to 0%. All animals exhibited weight gain by postoperative day 14. However, no statistically significant differences in body weight evolution were observed between the study groups, as represented in Figure 1. Resumption of intestinal transit occurred on average at postoperative day 1.7 ± 0.34 in Group A, 1.9 ± 0.22 in Group B, and 1.8 ± 0.30 in Group C, with no statistically significant differences observed between the groups. All abdominal wounds healed appropriately without postoperative complications.

### 3.2. Adhesion Formation

Adhesion scores were compared between groups using one-way ANOVA with Tukey’s post hoc correction for multiple comparisons.

**Figure 2:**
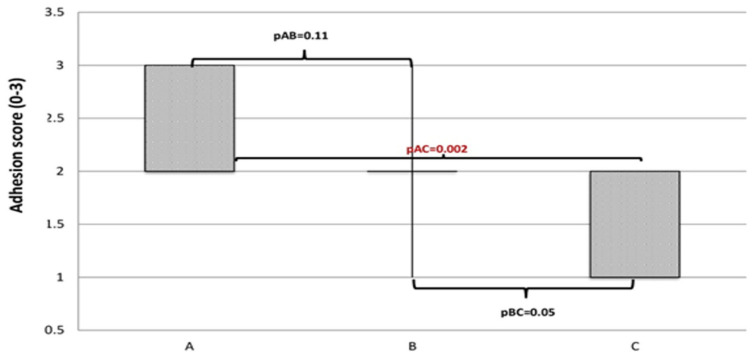
Severity of postoperative adhesion formation (assessed using a semi-quantitative scale ranging from 0 to 3). pAB = 0.11, pAC = 0.002, and pBC = 0.05 represent the *p*-values obtained from the comparative statistical analysis between groups A and B, groups A and C, and groups B and C, respectively. Data are presented as mean ± SD. Statistical analysis was performed using one-way ANOVA with Tukey’s post hoc test.

**Figure 3:**
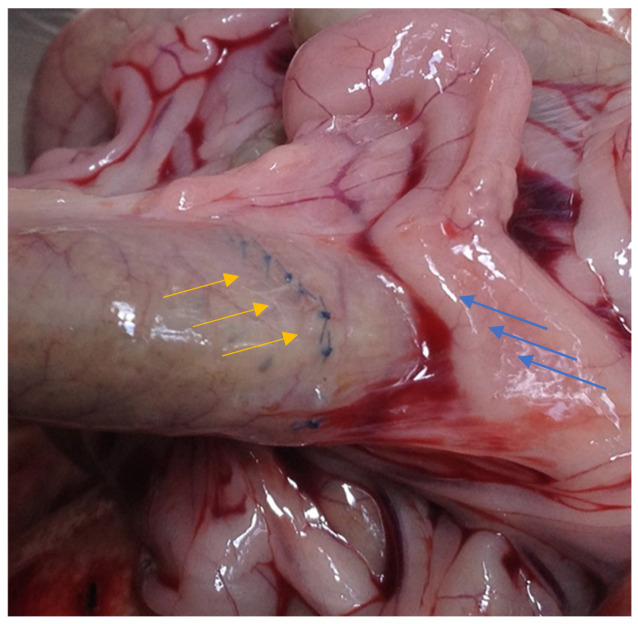
Representative image of limited adhesion formation (blue arrows) surrounding the anastomotic site (yellow arrows) in Group B.

The control group showed a more extensive adhesion formation involving intestinal loops and the omentum. However, these adhesions were easily lysed during surgical exploration. In Group C, the adhesion index was significantly lower (1.7 ± 0.15; Figure 2) compared with Group A (2.4 ± 0.16; *p* = 0.002) and Group B (2.1 ± 0.17; *p* = 0.05; Figure 3).

### 3.3. Histological Findings

Statistical analysis was performed using one-way ANOVA followed by Tukey’s post hoc test.

All anastomoses healed without obstruction or severe necrosis. The residual glandular epithelium did not show rejection in any of the cases. Regarding the mucosa, each sample revealed a mild increase in the number of mononuclear cells in the lamina propria with no ulceration in any of the cases taken in the study.

**Figure 4:**
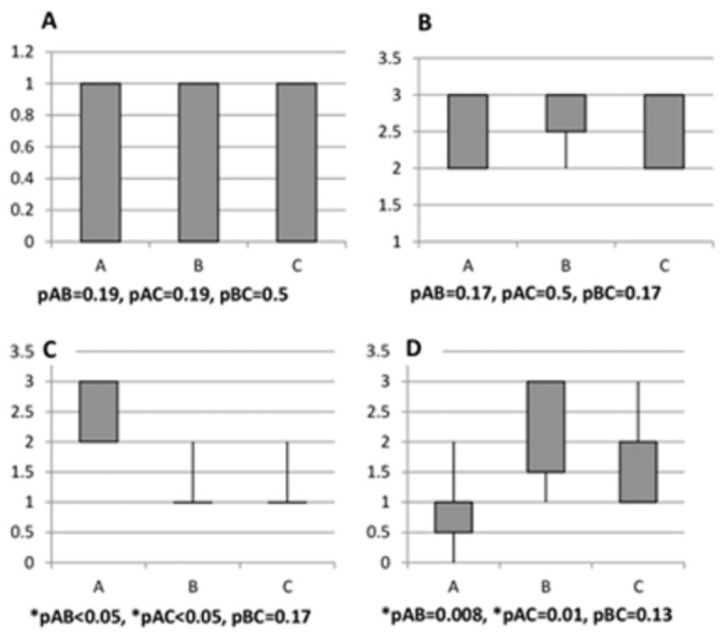
Histological scores for each evaluated parameter, graded on a semi-quantitative scale from 0 to 3: image (**A**)—necrosis; image (**B**)—epithelialization; image (**C**)—inflammation; image (**D**)—collagen deposition. pAB, pAC, and pBC indicate the *p*-values for comparisons between groups A vs. B, A vs. C, and B vs. C, respectively. Panel labeling and statistical annotations were standardized for clarity. Statistical analysis was performed using one-way ANOVA with Tukey’s post hoc test. Statistically significant results (*p* < 0.05) are indicated by an asterisk (*).

Necrosis was limited and scored similarly in the 3 groups, without evidence of any statistical difference after MSC injection (Table 1, Figure 4A, *p* > 0.05).

The histological analysis demonstrated complete epithelialization in the 3 groups, with intact submucosal and muscular layers, with no difference between the groups (Table 1, Figure 4B, *p* > 0.05).

In all 3 groups, histological analysis revealed an inflammatory reaction around the suture threads that was more pronounced in group A (granulomatous reaction and a severe inflammatory infiltrate with macrophages, giant cells, lymphocytes and neutrophils) compared with the MSC transplantation groups (Table 1, Figure 4C). Perianastomotic and intraperitoneal injection of MSCs caused a marked reduction in the inflammatory response with rare lymphocytes and neutrophils, with no significant difference between groups B and C (Figure 4C).

Histological analysis of intestinal specimens demonstrated collagen deposition at the anastomotic site, predominantly within the serosal layer. In Groups B and C, collagen deposition scores were significantly higher compared with the control group (Table 1, Figure 4D), indicating that MSC administration enhances collagen formation at the anastomotic level.

### 3.4. Hydroxyproline Content

Hydroxyproline was analyzed as a biochemical endpoint using one-way ANOVA followed by Tukey’s post hoc test.

Hydroxyproline levels were significantly increased following MSC administration in comparison with control animals, as represented in Table 2. Perianastomotic administration led to higher hydroxyproline content (0.831 ± 0.02 μg/mg tissue) compared with intraperitoneal administration (0.54 ± 0.02 μg/mg tissue), both significantly higher than the control group (0.251 ± 0.006 μg/mg tissue; *p* < 0.05 for all comparisons).

## 4. Discussion

### 4.1. Main Findings and Comparison with the Literature

MSCs play an important plastic role due to the secretion of growth factors, cytokines, and chemokines. These substances facilitate migration to the site of injury, exert an immunomodulatory effect, and enhance angiogenesis and stem cell differentiation. In vivo studies have found that MSC implantation directly into injured tissue has significant benefits [17], accelerating many parameters of healing.

MSCs can be easily isolated from bone marrow, peripheral blood, or adipose tissue. The advantages of extracting them from adipose tissue are numerous: low invasiveness and a quick and relatively inexpensive method [18].

In the literature, there are only three papers studying the involvement of MSC transplantation in the healing of gastrointestinal anastomoses in rat models [9,10,19]. Due to heterogeneity related to the number of subjects per group, method of administration, and the follow-up period, the results cannot be compared, and they are contradictory.

This study demonstrated histological and biochemical changes associated with MSC administration in colonic anastomoses. To the best of our knowledge, this is among the few studies providing a detailed analysis of anastomotic healing on postoperative day 14 after MSC administration.

Although we could not demonstrate that MSC transplantation decreases the incidence of anastomotic leakage, the rat experimental model allowed the assessment of colonic anastomotic healing in terms of histological features and hydroxyproline content. We developed an experimental model that can be used for further studies comparing different methods for improving anastomotic healing. We consider it inexpensive, reliable, technically simple, and based on an objective assessment of the healing process.

In our study, the 0% mortality rate and absence of anastomotic leakage can be explained by the fact that anastomoses performed under non-ischemic conditions in rat experimental models are close to optimal, with a low rate of complications when a proper technique is applied. However, the absence of clinically relevant adverse outcomes, together with the small sample size, limits the ability to draw conclusions regarding clinical efficacy.

In ischemic rat models, adipose tissue-derived MSCs resulted in less overall weight loss when animals were sacrificed on postoperative day 7 [10]. In our study, the differences between groups were not statistically significant; on postoperative day 14, all subjects exhibited weight gain. This suggests that the beneficial effect may be more pronounced in the early postoperative period and is not sustained over time.

Our study demonstrated a reduction in adhesion severity after intraperitoneal administration of MSCs. Similar results [11] were obtained in rat experimental models using biosutures (sutures coated with adipose tissue-derived MSCs). A possible explanation for these findings is that adhesion formation is also a consequence of tissue injury. Mesothelial cells play a key role in this process by producing a phospholipid-based surfactant that lubricates the viscera, protects against thrombosis, and secretes cytokines [20]. When tissues are injured, mesothelial cells activate the coagulation cascade, leading to fibrin deposition, while inflammatory cells (PMNs, macrophages, and lymphocytes) infiltrate the area [21]. MSC transplantation may provide a source of mesothelial progenitor cells that contribute to healing [20]. In addition, MSCs reduce the local inflammatory response. The effect on adhesion severity may be related to the broader distribution of MSCs within the peritoneal cavity following intraperitoneal administration, resulting in a more global modulation of peritoneal inflammation and fibrin formation compared to the more localized perianastomotic injection.

Assessment of anastomotic healing depends on histological features. Healing is influenced by the intensity of the primary inflammatory response, the rate of mucosal re-epithelialization, the amount, strength, and maturation of newly formed collagen, and the presence of collagenolysis during the early post-anastomotic period [13]. If the inflammatory response persists beyond the initial phase of healing (days 1–5), collagen formation becomes insufficient to ensure adequate anastomotic strength, increasing the risk of complications.

In our study, necrosis, epithelialization, inflammation, and collagen deposition were evaluated. No significant differences were observed between groups regarding necrosis and epithelialization, indicating that appropriate surgical technique with gentle tissue handling ensures favorable outcomes in this regard. We demonstrated that perianastomotic or intraperitoneal MSC administration increases collagen deposition and reduces the inflammatory response. These effects may contribute to stronger anastomoses and potentially reduce the risk of anastomotic leakage. These findings should be interpreted as exploratory, given the limited sample size and the absence of differences in clinically relevant outcomes.

When comparing histological parameters, the inflammatory response was reduced following MSC transplantation, without statistically significant differences (*p* > 0.05) between the groups receiving perianastomotic and intraperitoneal administration. In healthy tissue, injury activates immune cells that release pro-inflammatory mediators such as TNF-α, chemokines, leukotrienes, and free radicals. These mediators initiate inflammation, which is a physiological response to tissue injury. After clearance of necrotic tissue, immune cells become redundant; however, their prolonged activation may impair proper healing [22]. MSC administration reduces cytokine synthesis and upregulates anti-inflammatory molecules in rats, exerting an immunomodulatory effect [23]. Several studies have demonstrated that MSCs are strongly immunosuppressive both in vivo and in vitro [18,22].

Collagen deposition was increased following MSC transplantation. A possible explanation for this effect is related to another mechanism of MSCs in tissue repair. Experimental studies have shown that, in MSC-mediated healing, differentiation into damaged cells is a rare event. Instead, MSCs promote the regenerative potential of host stem cells and enhance fibroblastic activity [9,23]. It is well-known that fibroblasts are the main effector cells in tissue repair, being responsible for extracellular matrix production and tissue remodeling [22] and the stimulation of fibroblastic activity leads to increased collagen synthesis [9,24,25,26].

Hydroxyproline levels, as an indicator of collagen content, were determined as an objective parameter for assessing healing and anastomotic strength [13]. However, these parameters reflect indirect biochemical and structural markers of healing and do not provide direct evidence of mechanical strength of the anastomosis, as no bursting pressure or tensile strength testing was performed. The results obtained suggest that MSC transplantation is associated with enhanced histological and biochemical markers of anastomotic healing. When comparing collagen deposition and hydroxyproline content, significantly higher values were observed after perianastomotic administration compared with intraperitoneal administration. This finding suggests that direct perianastomotic delivery may provide a more targeted local effect on fibroblast activation and collagen synthesis at the anastomotic site.

It is plausible that the reduction in the inflammatory response, together with the increase in collagen content, may contribute to improved mechanical strength of digestive anastomoses.

The use of MSC transplantation as an adjuvant strategy to improve anastomotic healing may be considered a feasible approach. Potential disadvantages include the time required for cell preparation (limiting its use mainly to elective procedures), the need for trained personnel in cell therapy techniques, and appropriate storage and handling conditions due to the use of viable cells. However, several factors support its clinical potential for anastomotic leak prevention, including minimally invasive harvest, compatibility with both open and laparoscopic surgery, single-dose intraoperative administration, additional beneficial effects such as reduction in postoperative adhesions, and the absence of major ethical concerns.

Further studies are required to evaluate the effects of MSCs at different postoperative time points (both short-term and long-term outcomes) and to identify agents that are capable of enhancing their therapeutic efficacy.

### 4.2. Clinical Implications and Study Limitations

The results of the present study suggest that MSC therapy may represent a promising adjuvant strategy associated with improved histological and biochemical markers of colonic anastomotic healing. The observed improvement regarding histological parameters (particularly increased deposition of collagen and reduced inflammatory response), together with the beneficial effect on adhesion formation, supports the potential translational value of MSC-based approaches in gastrointestinal surgery.

However, it must be emphasized that the present study used a non-ischemic colonic anastomosis model, which does not fully reproduce the multifactorial pathophysiology of clinically relevant anastomotic leakage, and therefore, translational extrapolation should be made with caution.

From a clinical perspective, MSC administration could be particularly useful in elective colorectal procedures, where preoperative preparation and intraoperative cell application are feasible. The single-dose administration strategy and compatibility with both open and minimally invasive surgical techniques further justify the potential applicability of this approach. In addition, the observed decrease in postoperative adhesion formation may represent an additional advantage, perhaps decreasing long-term morbidity related to adhesive disease.

However, several limitations must be acknowledged. First, the experimental design was based on a non-ischemic colonic anastomosis model, which does not fully reproduce the complex pathophysiological conditions associated with anastomotic leakage in clinical settings. Second, the relatively small sample size might limit the statistical power of certain comparisons. Third, the follow-up period of 14 days does not allow for evaluating long-term outcomes or late complications. Nevertheless, no mechanical strength testing of the anastomosis was performed, which could have provided additional functional information concerning tissue integrity. Another limitation of the current study is attributed to the absence of flow-cytometric analysis for MSC surface markers in accordance with full ISCT criteria [12], which would have provided more comprehensive phenotypic characterization of the isolated cells. Moreover, no direct mechanical testing of anastomotic integrity (such as bursting pressure or tensile strength assessment) was performed in this study. Therefore, mechanical strength of the anastomosis cannot be inferred from the present results.

Finally, even though MSC therapy demonstrated clear biological effects in this experimental setting, translational application requires further investigation regarding optimal dosing, timing, route of administration, and standardization of cell preparation protocols. Additional studies are also required to evaluate the long-term safety and efficacy of this approach, as well as its potential synergy with other therapeutic strategies aimed at improving anastomotic healing.

## 5. Conclusions

MSC transplantation was associated with improved histological and biochemical markers of colonic anastomotic healing in a non-ischemic rat model. These effects may contribute to improved tissue integrity at the anastomotic site and may potentially reduce postoperative complications such as adhesion formation.

Taken together, these findings support the potential role of MSC-based therapy as an adjuvant strategy in gastrointestinal surgery. However, further experimental and clinical studies are necessary to better define the optimal route of administration, dosing, timing, and long-term effects, as well as to confirm its efficacy and safety in clinically relevant ischemic models and human patients.

## Figures and Tables

**Table 1:** Mean histological scores (semi-quantitative scale 0–3).

Outcome, Mean (SD)	Group		
	A	B	C
Necrosis	0.6 (0.16)	0.4 (0.16)	0.4 (0.16)
Epithelialization	2.6 (0.16)	2.8 (0.13)	2.6 (0.16)
Inflammation	2.6 (0.16)	1.2 (0.13)	1.4 (0.16)
Collagen deposition	1 (0.21)	2.2 (0.24)	1.8 (0.24)

Data are presented as mean (SD). Statistical analysis was performed using one-way ANOVA followed by Tukey’s post hoc test.

**Table 2:** Hydroxyproline content in colonic anastomotic tissue.

Group	Hydroxyproline (μg/mg Tissue)
A (Control)	0.251 ± 0.006
B (Perianastomotic MSCs)	0.831 ± 0.02
C (Intraperitoneal MSCs)	0.54 ± 0.02

Data are presented as mean ± SD.

## Data Availability

The original contributions presented in this study are included in the article. Further inquiries can be directed to the corresponding authors.
